# Bovine Tuberculosis Antemortem Diagnostic Test Agreement and Disagreement in a Naturally Infected African Cattle Population

**DOI:** 10.3389/fvets.2022.877534

**Published:** 2022-07-07

**Authors:** Robert F. Kelly, Lina Gonzaléz Gordon, Nkongho F. Egbe, Emily J. Freeman, Stella Mazeri, Victor N. Ngwa, Vincent Tanya, Melissa Sander, Lucy Ndip, Adrian Muwonge, Kenton L. Morgan, Ian G. Handel, Barend M. de C. Bronsvoort

**Affiliations:** ^1^The Royal (Dick) School of Veterinary Studies and Roslin Institute, University of Edinburgh, Edinburgh, United Kingdom; ^2^School of Life Sciences, University of Lincoln, Lincoln, United Kingdom; ^3^School of Veterinary Sciences, University of Ngaoundere, Ngaoundere, Cameroon; ^4^Cameroon Academy of Sciences, Yaoundé, Cameroon; ^5^Tuberculosis Reference Laboratory Bamenda, Hospital Roundabout, Bamenda, Cameroon; ^6^Laboratory of Emerging Infectious Diseases, University of Buea, Buea, Cameroon; ^7^Institute of Ageing and Chronic Disease and School of Veterinary Science, University of Liverpool, Neston, United Kingdom

**Keywords:** bovine tuberculosis, diagnostic test performance, cattle, epidemiology, Africa, *Mycobacterium bovis*

## Abstract

The interferon-gamma (IFN-γ) assay and single comparative cervical skin test (SCITT) are used to estimate bovine tuberculosis (bTB) prevalence globally. Prevalence estimates of bTB, caused by *Mycobacterium bovis*, are poorly quantified in many Sub-Saharan African (SSA) cattle populations. Furthermore, antemortem diagnostic performance can vary at different stages of bTB pathogenesis and in different cattle populations. In this study, we aim to explore the level of agreement and disagreement between the IFN-γ assay and SCITT test, along with the drivers for disagreement, in a naturally infected African cattle population. In, 2013, a pastoral cattle population was sampled using a stratified clustered cross-sectional study in Cameroon. A total of 100 pastoral cattle herds in the North West Region (NWR) and the Vina Division (VIN) were sampled totalling 1,448 cattle. Individual animal data and herd-level data were collected, and animals were screened using both the IFN-γ assay and SCITT. Serological ELISAs were used to detect exposure to immunosuppressing co-infections. Agreement analyses were used to compare the performance between the two bTB diagnostic tests, and multivariable mixed-effects logistic regression models (MLR) were developed to investigate the two forms of IFN-γ assay and SCITT binary disagreement. Best agreement using the Cohen's κ statistic, between the SCITT (>2 mm) and the IFN-γ assay implied a ‘fair-moderate' agreement for the NWR [κ = 0.42 (95%CI: 0.31–0.53)] and ‘poor-moderate' for the VIN [κ = 0.33 (95% CI: 0.18–0.47)]. The main test disagreement was the animals testing positive on the IFN-γ assay and negative by the SCITT. From MLR modeling, adults (adults OR: 7.57; older adults OR = 7.21), females (OR = 0.50), bovine leucosis (OR = 2.30), and paratuberculosis positivity (OR = 6.54) were associated with IFN-γ-positive/SCITT-negative disagreement. Subsets to investigate diagnostic test disagreement for being SCITT-positive and IFN-γ-negative also identified that adults (adults OR = 15.74; older adults OR = 9.18) were associated with IFN-γ-negative/SCITT-positive disagreement. We demonstrate that individual or combined use of the IFN-γ assay and SCITT can lead to a large variation in bTB prevalence estimates. Considering that animal level factors were associated with disagreement between the IFN-γ assay and SCITT in this study, future work should further investigate their impact on diagnostic test performance to develop the approaches to improve SSA prevalence estimates.

## Introduction

Bovine tuberculosis is a chronic disease of cattle, caused by *Mycobacterium bovis*, and is an important zoonosis associated with close interaction with cattle or consumption of raw dairy products (1). Close contact between cattle and people is commonplace in livestock rearing communities in Sub-Saharan Africa (SSA). Consequently, it is vital to understand the potential direct impacts of bTB on livestock health as well as its public health risks in such communities. A key metric in understanding bTB epidemiology is to quantify the burden of disease in cattle by estimating bTB prevalence. However, the prevalence estimates in the live animals rely on the use of diagnostic tests that are imperfect. Identifying characteristic TB lesion pathology has been used to describe the epidemiology of bTB in abattoirs in many African countries (2). Specificity of lesion detection is high (>95%) and can be improved further with culture from lesions or using molecular techniques such as PCR to characterise *M. bovis* bacilli (3, 4). However, identification of lesions is not a gold-standard diagnostic test, as its sensitivity can be low (28.5%), particularly in the early stages of infection (5) and can only be used in cattle postmortem.

Antemortem diagnostic tests for bTB are based upon measuring the specific aspects of the immune response to *M. bovis* antigens (2, 6, 7). *Mycobacterium bovis* infections predominately stimulate cell-mediated immunity (CMI) responses in cattle (8–10); therefore, antemortem diagnostic tests have tended to focus on detecting this CMI response in different formats, including the *in vivo* tuberculin skin tests known as the single intradermal test (SIT) and single comparative intradermal tuberculin test (SCITT) (2, 6, 11) and the *in vitro* IFN-γ assay. The SCITT is recognised by the World Organisation for Animal Health (OIE) as the primary diagnostic test for bTB diagnosis (3), with a very high specificity (median: 100%; CrI: 99–100%) (7, 12) but low sensitivity (median: 50.0%; CrI: 26–78%) reported in a recent extensive meta-analysis (13). As a result, there is an extremely high risk of infected animals testing negative (i.e., false-negative animals) resulting in gross underestimation of the bTB prevalence and, when used in control programmes, an increased risk of retaining positive animals that may continue to transmit within the herd or to naïve herds when animals are traded. The IFN-γ assay detects the predominant Th1 immune response to *M. bovis* and experimentally can detect positive animals from 2 weeks post-infection (14). Consequently, the IFN-γ assay is reported to have a higher sensitivity (median: 67%; CrI: 49–82%) than the SCITT with a slightly lower specificity (median: 98%; CrI: 96–99%) (13). In addition to diagnostic performance, the logistics involved in the use of either test is also important to appreciate when applied to a SSA context. The SCITT requires restraint of the animal and injection of the avian and bovine tuberculin intradermally in the neck. It has the drawbacks of requiring a repeat visit to remeasure the skin after 3 days and good handling facilities to assess skin thickness. This is particularly challenging in the SSA setting because of the absence of cattle identification systems in many SSA countries and the dangers when handling African cattle breeds that are predominately horned. The IFN-γ assay on the other hand requires a single blood sample that can be done from the tail vein. The disadvantage compared to the SCITT is that the IFN-γ assay does require local laboratory facilities to conduct the time-limited cell stimulation and incubation stages followed by the collection of the plasma and an ELISA.

The probability of a diagnostic test correctly classifying animals depends on its sensitivity, specificity, and the underlying true prevalence of the disease in the population. The SCITT and IFN-γ assays measure different aspects of the CMI response (15, 16) and therefore may not detect the same population of bTB-positive cattle. In addition, the performance of the two tests may vary between different cattle populations and, depending on the performance requirements for testing, can be adjusted through changing the diagnostic cut-off value for a positive of either test (16–19). Another approach is the so-called parallel testing, where the combination of being positive on one or both tests has been used to improve overall sensitivity of the testing system (20–22). It is important, given that the SCITT and IFN-γ assays detect the different aspects of the CMI response, to understand why these two tests disagree to better interpret their results in the field. Factors that may lead to test disagreement can be classified into those associated with laboratory method, operator error, or host-related factors. Understanding the impact of host-related factors is of particular interest to understand variation in test performance depending on animal's characteristics. For example, diagnostic tests could be selected, or their interpretation adjusted based on the signalment of individual animals. Host-related factors previously identified include animal age, breed, pregnancy status, and causes of immunosuppression such as co-infections (7). Results from a previous study in Cameroon found that co-infection with *Fasciola gigantica* reduced the sensitivity of the IFN-γ assay (23), and studies elsewhere highlight a similar impact on SCITT sensitivity with other *Fasciola* species (24). Other immunosuppressive co-infections may also impact bTB antemortem diagnostic test performance (25–27), such as immunosuppressing viruses or *Mycobacterium avium* subspecies *paratuberculosis* (MAP), which have been shown to lead to false-negative results (28). Identifying factors that influence IFN-γ assay and SCITT performance and where disagreement is occurring between tests will highlight the potential challenges using these tests and what adjustments may be needed to improve interpretation of the results.

In this study, we investigate the level of agreement between the IFN-γ assay and the SCITT and host-related factors associated with their diagnostic disagreement. We then compare the impact of their different performances on the prevalence estimates of bTB using the IFN-γ assay, the SCITT and the “parallel” test combination for pastoral cattle populations in Cameroon.

## Materials and Methods

### Study Design

**Figure 1:**
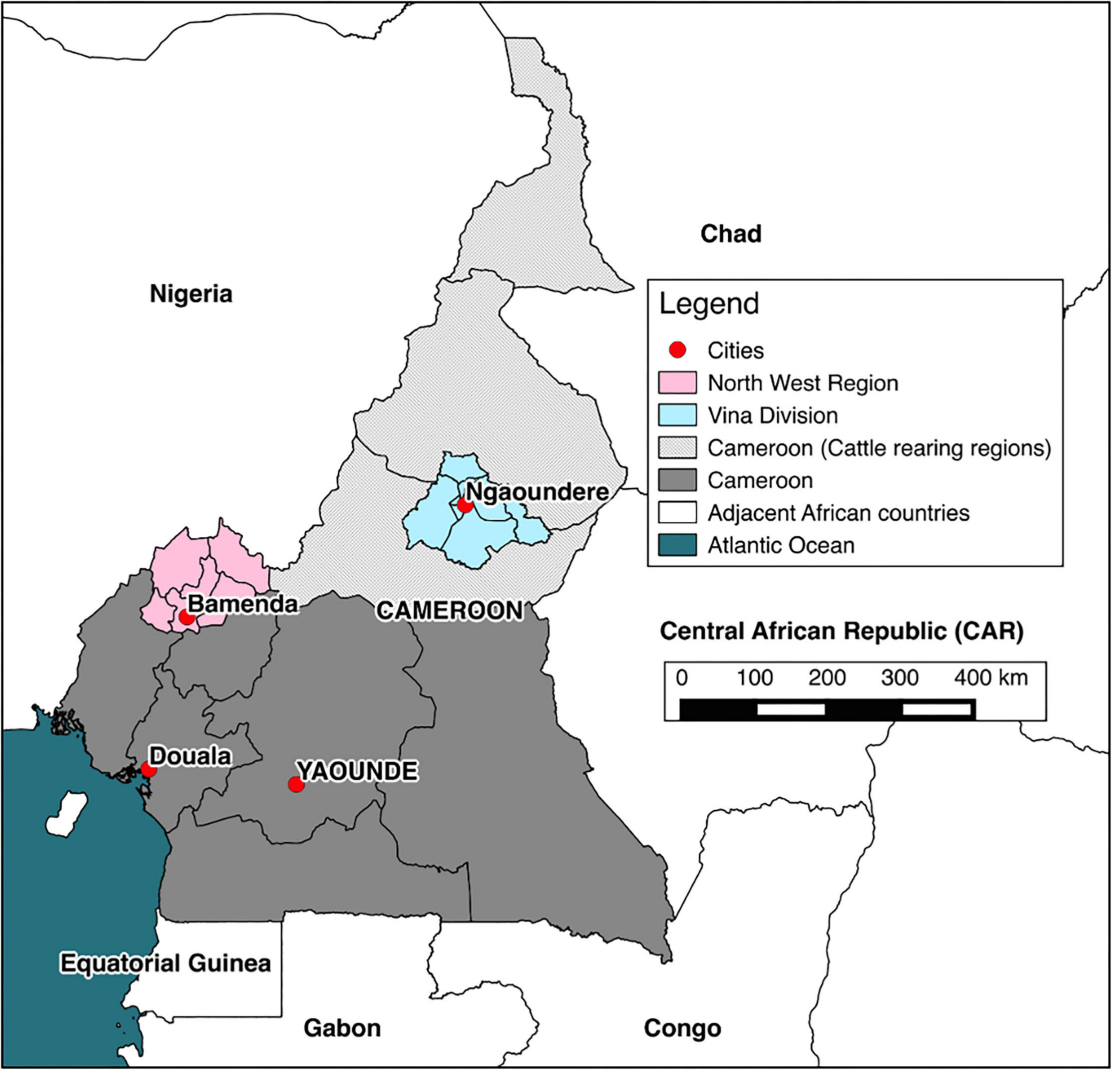
Map of Cameroon. The location of cattle rearing areas (light grey), study sites (pink and blue), and major cities (red).

The dataset used for this analysis was a part of a larger study investigating the epidemiology of bTB in Cameroonian cattle populations (29). The study sites were the Northwest Region (NWR) and the Vina Division (VIN) of the Adamawa Region (AR) of Cameroon (Figure 1), previously highlighted as important cattle keeping areas of Cameroon (30). A cross-sectional study was conducted, sampling two pastoralist populations (NWR & VIN) where *M. bovis* has been confirmed present (30, 31). Sampling was conducted between January–May 2013 in the NWR and September–November 2013 in the VIN, respectively. Pastoralist cattle populations in the NWR and VIN were estimated to be 506,548 and 176,257, respectively, from the Ministry of Livestock, Fisheries and Industrial Agriculture /Ministere de l'Elevage des Peches et Industries Animales (MINEPIA) vaccination records (32). The pastoralist cattle population eligible for sampling were herds listed in vaccination records at 81 ZVSCCs in the NWR and 31 ZVSCCs in the VIN in 2012. There were 5,053 cattle herds in the NWR and 1,927 in the VIN, with a range of 1–215 animals per herd. A population weighted stratified random sample of registered herds was sampled in each of the two study sites. The sample was stratified by sublocation within each administrative area; seven divisions in the NWR and eight subdivisions in the VIN. The number of herds sampled from each sublocation was proportional to the total number of herds within that sublocation. The sample size for this bTB focused project was based on an clustered random sample with an estimated animal level bTB prevalence of ~10%, a within herd variance of 0.15 and between herd variance of 0.01, an average herd size of 70, a relative cost of 12:1 for herd/animal and relative error of ± 15% (Survey Toolbox; AusVet) (33). This gave a target sample size of 15 cattle per herd and 88 herds under the simplifying assumption of perfect test performance. The final sample was 100 herds with 50 at each site (NWR and VIN). Within each herd, the 15 samples were stratified to each of three age classes: young (≥0.5 years and <2 years), adult (≥2 and <5 years), and old (≥5 years). If there were insufficient animals of one age group, additional animals of any age were sampled.

### Data Collection

Pastoral herds were visited and a local translator explained the project to the herdsman in either Foulfulde, Pidgin English, or French. Individuals were asked, in the language in which they were most comfortable, to give verbal consent to participate in the study. The same local translator, who was unaware of animal disease-status, selected 15 animals per herd at random. The animals were identified used disposable numbered Tyvek® wrist bands on the forelimb and horn on the side the SCITT would be conducted on. All sampled animals were then examined by the same veterinarian, with data recorded at an individual animal level to accompany samples. These included signalment [sex, age by dentition score (DS), breed, and body condition score (BCS)]. The method for ageing by DS and BCS was carried out on 5-point scales (34). “Improved” breed cattle were defined as cattle that had the phenotypic appearance of mixed *Bos indicus* breeding. “Exotic” cattle defined as those cattle that had the phenotypic appearance or which were reported by the farmer to be fully or mixed *Bos taurus*. Plain and heparinised blood samples were collected from the jugular or tail vein. All information was initially recorded on paper forms, which were later transferred into a relational Access database (Microsoft Access®). Unless otherwise stated, all animals sampled were tested using all infectious disease diagnostic tests.

### Diagnostic Tests for Bovine Tuberculosis and Other Infectious Diseases

#### IFN-γ Assay

For the IFN-γ assay (20), three aliquots of 1.5 ml of heparinised blood per animal were stimulated with either 15 μl of avian PPD, bovine PPD (Prionics® Lelystad Tuberculin PPD), or PBS and then incubated for 24 h at 37°C within 8 h of sampling (usually <3 h). Incubated blood was centrifuged at 300 g for 10 min, and then, three plasma aliquots per animal were stored at −20°C in a portable travel freezer. Electrical supplies were maintained by mains electricity, portable generators, or from vehicle batteries where necessary in the field. Plasma samples were transported at −20°C to Laboratory of Emerging Infectious Diseases (LEID), University of Buea, Cameroon, where the IFN-γ assay ELISA was conducted. Prior to starting the protocol, reagents were reconstituted and samples were allowed to reach room temperature (22 +/-5°C). The avian PPD, bovine PPD, and PBS-stimulated plasma samples were diluted 1:1 with dilution buffer. Diluted plasma samples were added to the pre-coated 96 well plate along with duplicates of kit positive, negative, and PBS controls. The 96 well plate was incubated on a microplate shaker, at 600 rpm for 60 min at 22 +/-5°C, and once complete, the plate washed 6 times with wash buffer. About 100 μl of conjugate was added to the 96 well plate incubated for 60 min and washed as previous. About 100 μl of enzyme substrate was added to the 96 well plate incubated for 30 min as previous in the dark. Finally, 50 μl of stopping solution was then added to the 96 well plate and read at 450 nm using an automated microplate reader (Thermoscientific® Multiskan Go). For interpretation, PBS control ODs were subtracted from corresponding sample ODs. The acceptable averaged negative bovine was <0.13 and positive bovine control was >0.70. For each sample, the difference in OD of the sample stimulated with bovine PPD minus the mean OD of the avian PPD was calculated for interpretation. At standard interpretation, as per commercial kit instructions, animals with a bovine PPD plasma sample of ≥0.1 that of avian PPD and PBS were classed as *M. bovis* infected.

#### Single Cervical Intradermal Tuberculin Test (SCITT)

All SCITTs were performed and interpreted by the same experienced veterinarian (RK) following the standard protocol used in the United Kingdom. At the time of sampling, each animal was appropriately restrained by either casting on the ground or tied up to trees using ropes (Day 0). Left or right side of the cervical neck was used, depending upon accessibility, and ID bands were placed on the leg and/or horn on the same side of the animal. Two areas, approximately 12 cm apart, in the mid-cervical neck had the hair clipped (using scissors) to mark the injection sites. The skin thickness was measured using skin callipers and recorded for each site prior to injection. A total of two multidose automatic syringes (McLintock®) were used to inject 0.1 ml of avian and bovine PPD (Prionics® Lelystad Tuberculin PPD) intradermally in the dorsal and ventral sites, respectively. The injection site was palpated to confirm whether PPDs were injected intradermally. Multidose automatic syringe needles were swabbed with surgical spirit between individual cattle. On a return visit, approximately 72 h later (Day 3), the skin thickness of the two injection sites was remeasured using skin callipers. The difference in bovine and avian PPD skin measurements was calculated for each bovine to determine whether the animal is infected with *M. bovis*. First, the difference between the skin measurement of PPD injection sites on days 0 and 3 was calculated. Then, the difference between bovine and avian was calculated:

- Avian skin reaction difference (A) = skin thickness day 3 – skin thickness day 0 (mm)
- Bovine skin reaction difference (B) = skin thickness day 3 – skin thickness day 0 (mm)
- PPD skin reaction difference = B–A

Second, clinical signs at the injection site (e.g., oedema) were recorded. The results for each animal were then interpreted at two cut-off values. At severe interpretation, animals with a PPD skin reaction difference of >2.0 mm +/- clinical signs at the injection site indicated the presence of *M. bovis* infection. At standard interpretation, a PPD skin reaction difference of >4.0 mm +/- clinical signs at the injection site indicated the presence of *M. bovis* infection. These two cut-off values were then evaluated in subsequent agreement analysis.

#### Serum Antibody ELISAs

After collection, all serum samples were heat treated at 56°C for 120 min and stored at −20 ^o^C until tested. Serum samples were then tested for exposure to various infections using serological ELISAs. For the *F. gigantica* antibody ELISA (35), immulon-2 ELISA 96-well plates were coated with 100 μl of 1 μg/ml *F. gigantica* E/S antigen in 0.1 M carbonate buffer (pH 9.6). Plates were incubated for 1 h at room temperature and then refrigerated at 2–4°C overnight. Plates were then washed six times (two short washes and one 5-min wash repeated for two times) with pH 7.2 PBS containing 0.05% Tween-20 (PBS-Tween). Each well was blocked with 200 μl of blocking buffer for 1 h at 37°C [4% skimmed powder (Marvel, Premier International Foods®, Spalding UK)] in PBS-Tween. Plates were washed six times, and 100 μl of sera diluted 1:200 in blocking buffer was added to each well. Positive and negative serum controls were added to the plate in duplicate, at the same concentration as the test sera, and incubated at 37°C for 1 h. The plates were again washed, 100 μl of 1:1500 mouse anti-bovine IgG HRP conjugate (Serotec®, UK) in blocking buffer was added, and then, the plates were incubated at 37^O^C for 1 h. After washing, 100 μl of TMB substrate (acetate buffer pH 5 and tetramethylbenzidine in a methanol-based solution, MAST Diagnostics, Bootle, Merseyside, UK) was added and incubated at room temperature for 20 min in the dark. Finally, 100 μl of stopping solution (10% hydrochloric acid) was added and the colour change measured at 450 nm using an automated microplate reader (Thermoscientific® Multiskan Go). The results were obtained as an optical density (OD) and expressed as a percent positivity value (PPV). For this study, samples PPV <23.4% are considered negative and PPV ≥23.4% are considered positive (35).

Screening for MAP exposure was conducted using the ID.vet Screen® Paratuberculosis Serum Indirect Multi-species ELISA (36). In brief, 10 μl of each serum sample was diluted to 1:10 with the provided dilution buffer. The diluted sample was added to a well on the purified MAP antigen coated microplate and incubated at 21°C for 45 min. The microplate was washed three times with 300 μl of wash solution, and 100 μl of supplied conjugate was added for 30 min at 21°C. Using the same method, the microplate was washed again, and 100 μl of substrate solution was added to each well for 15 min at 21°C. To stop the reaction, 100 μl of stop solution was added to each well and the microplate was read at 450 nm. For a microplate to be considered valid, the mean of the duplicate positive (PC) and negative (NC) controls was calculated. For a microplate to pass, mean PC optical density (OD) needed to be > 0.35 and the mean positive and negative OD ratio (ODPC/ ODNC) needed to be >3. For each sample, the sample to positive ratio (S/P%) was calculated by (ODsample–ODNC) / (ODPC -ODNC) X 100. The manufacturers suggest that samples S/P% ≤ 60% are considered negative; S/P% >60% and <70% are considered doubtful; and S/P% ≥70% are considered positive. For this study, samples S/P% <70% are considered negative and S/P% ≥70% are considered positive.

Screening for bovine leukaemia virus (BLV) exposure was conducted using the ID Screen® BLV Serum Competitive ELISA (37). In brief, 25 μl of each serum sample was diluted to 1:4 with the provided dilution buffer. The diluted sample was added to a well on the BLV gP51 antigen-coated microplate and incubated at 21°C for 45 min. The microplate was washed three times with 300 μl of wash solution, and 100 μl of supplied conjugate was added for 30 min at 21°C. Using the same method, the microplate was washed again, and 100 μl of substrate solution was added to each well for 15 min at 21°C. To stop the reaction, 100 μl of stop solution was added to each well, and the microplate was read at 450 nm. For a microplate to be considered valid, the mean of the duplicate PC and NC was calculated. For a microplate to pass, mean NC OD needed to be > 0.7 and the mean positive and negative OD ratio (ODPC/ ODNC) needed to be >0.3. For each sample, competition percentage (S/N%) was calculated by (ODsample / ODNC) x 100. The manufacturers suggest that samples S/N% ≤ 50% are considered positive; S/N% >50% and <60% are considered doubtful; and S/N% ≥60% are considered negative. For this study, samples S/N% ≤ 50% are considered positive and S/N% >50% are considered negative.

Screening for bovine viral diarrhoea virus (BVDV) exposure was conducted using the ID Screen® BVDV Serum Competitive ELISA (38). In brief, 15 μl of each serum sample was diluted to 1:9 with the provided dilution buffer. The diluted sample was added to a well on the BVDV p 80 antigen-coated microplate and incubated at 37°C for 45 min. The microplate was washed three times with 300 μl of wash solution, and 100 μl of supplied conjugate was added for 30 min at 21°C. Using the same method, the microplate was washed again and 100 μl of substrate solution was added to each well for 15 min at 21°C. To stop the reaction, 100 μl of stop solution was added to each well and the microplate was read at 450 nm. For a microplate to be considered valid, the mean of the duplicate PC and NC was calculated. For a microplate to pass, mean NC OD needed to be > 0.7 and the mean positive and negative OD ratio (ODPC/ ODNC) needed to be >0.3. For each sample, competition percentage (S/N%) was calculated by (ODsample / ODNC) x 100. The manufacturers suggest that samples S/N% ≤ 40% are considered positive; S/N% >40% and ≥50% are considered doubtful; and S/N% >50% are considered negative. For this study, samples S/N% ≤ 40% are considered positive and S/N% >40% are considered negative.

### Statistical Analyses

All statistical analyses were performed using packages and functions in R version 3.6.1 (39). Graphics were produced using the *ggplot2* package (40). Spatial data were displayed using QGIS 2.2® (41) or *tidyverse* collection of R packages (42) and shapefiles obtained from the GADM database of Global Administrative Areas (www.gadm.org).

#### Bovine Tuberculosis Diagnostic Test Agreement

Agreement analyses were in this study to compare the performance between diagnostic as no gold-standard diagnostic tests were available. Agreement was defined as how much two diagnostic tests, which measure the same response, agree with each other (43). In the absence of gold-standard diagnostics, agreement analysis is used in this study to explore the positive cut-off values for the SCITT with continuous results for both tests to be converted to categorical results (e.g., positive and negative). For the IFN-γ assay, ≥0.1 was used as the positive cut-off value compared to the SCITT at >2 mm and >4 mm cut-off by study site. Percentage agreement and Cohen's κ statistic were used to quantify binary agreement between two tests. The agree, *kappa2*, and *rater.bias* functions in the *irr* package (44) were used to calculate percentage agreement and Cohen's κ statistic. Percentage agreement was used as a provisional measure of agreement between two diagnostic tests and was calculated as follows (43):


$$\begin{array}{l} \text{Percentage agreement or observed agreement (OP)}\\ \;=\left(a+d\right)/\left(a+b+c+d\right)\times \;100 \end{array}$$

Percentage agreement does not distinguish agreement between positive or negative diagnostic test results and does not adjust for chance. Consequently, the Cohen's κ statistic was also calculated to determine the level of agreement, beyond chance, between two categorical diagnostic tests (e.g., positive or negative) (45):


$$\begin{array}{l} \text{Expected agreement (EP)}\\ \;=\left[\left(\left(a+b\right)\left(a+c\right)\right)/n+\left(\left(c+d\right)\left(b+d\right)\right)/n\right]/n\\ \text{Cohen's}\kappa \text{statistic}=\left(OP-EP\right)/\;\left(1-EP\right) \end{array}$$

Cohen's κ statistic was interpreted as =1 (perfect agreement), 0.81–1 (almost perfect agreement), 0.61–0.8 (substantial agreement), 0.41–0.6 (moderate agreement), 0.21–0.4 (fair agreement), 0.01–0.2 (poor agreement), and ≤ 0 (no agreement) (46). The effect of very low or high prevalence is thought to have a negligible effect on the calculation (44, 47).

#### Prevalence Estimates

The structure of the cross-sectional study design was incorporated into analyses using the *svydesign, confint*, and *svyby* functions in the *survey* package (48). For estimating bTB prevalence using SCITT, IFN-γ, and combined tests, true prevalence (43) was estimated using the epi.prev function in the epiR package (49) for pastoral cattle population by study site. Specific differences in sample statistics were identified by non-overlapping confidence intervals (CI) at 95% level (50).

#### Risk Factors Associated With Diagnostic Test Disagreement

**Figure 2:**
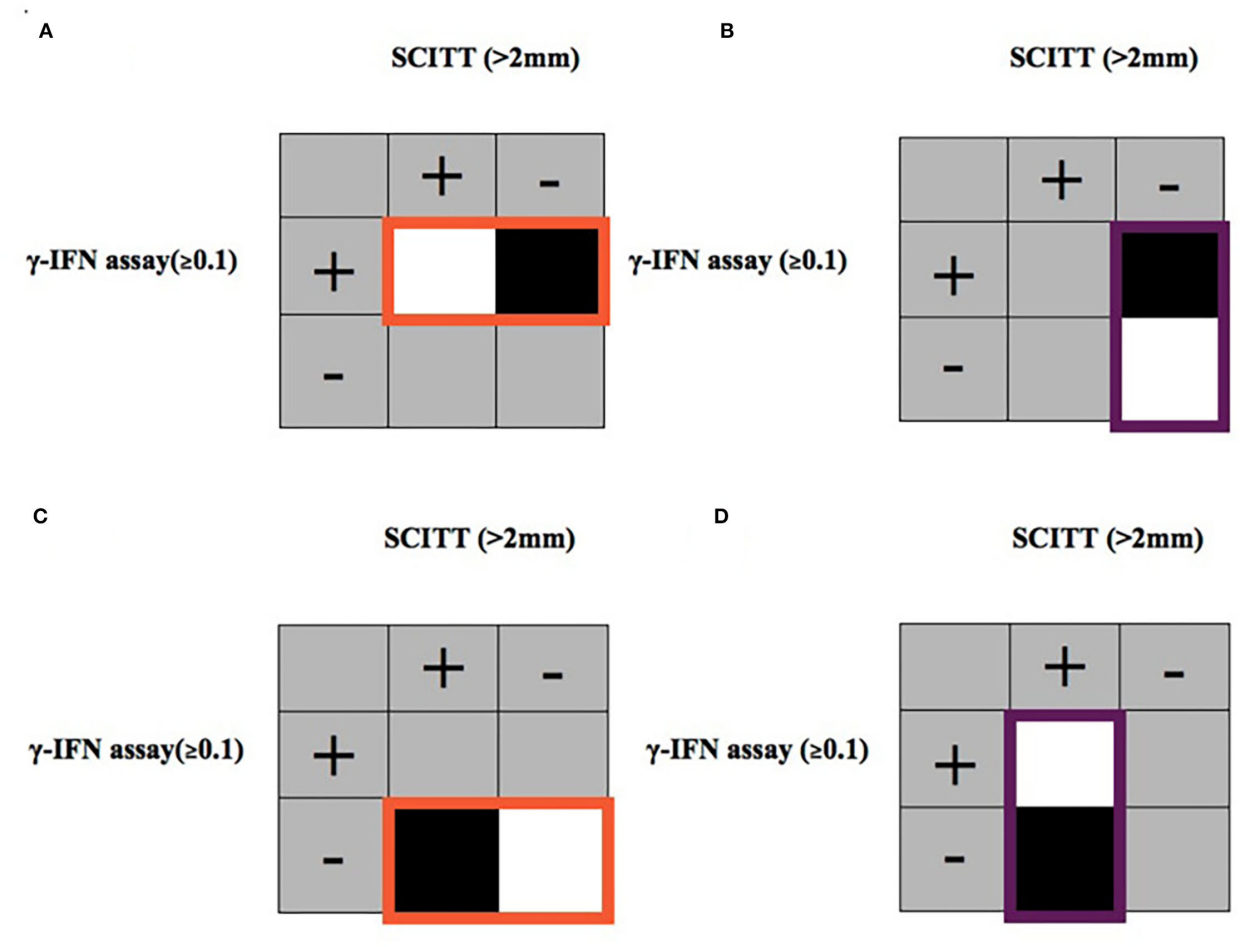
Diagrammatic representation of data subset to investigate two types of test disagreement. 1. IFN-γ assay-positive (≥0.1) and SCITT-negative (≤ 2 mm) using models **(A,B)**. Model **(A)** Subset of all cattle with a IFN-γ assay-positive response highlighted in orange with SCITT as the dependent variable. Model **(B)** Subset of all cattle with a SCITT-negative response highlighted in purple with IFN-γ assay as the dependent variable. Black areas indicate “positive” (disparate results) and white areas indicates “negative” (agreeing results) of the dependent variable. 2. IFN-γ assay-negative (<0.1) and SCITT-positive (>2 mm) in pastoral cattle using models **(C,D)**. Model **(C)** Subset of all cattle with a IFN-γ assay-negative response highlighted in orange with SCITT as the dependent variable. Model **(D)** Subset of all cattle with a SCITT-positive response highlighted in purple with IFN-γ assay as the dependent variable. Black areas indicate “positive” (disparate results) and white areas indicates “negative” (agreeing results) of the dependent variable.

Multivariable mixed-effects logistic regression (MLR) models were built to investigate the two forms of IFN-γ assay and SCITT binary disagreement using a combined dataset from both study sites, subsetted by the two forms of diagnostic disagreement:

1. IFN-γ assay-positive and SCITT-negative (Figure 2).
a. Model a): Subset of IFN-γ assay (≥0.1)-positive animals, using SCITT (≤ 2 mm) status as the dependent variable (IFN-γ/SCITT: +/- = 1; +/+ = 0).
b. Model b): Subset of SCITT (≤ 2 mm)-negative animals, using IFN-γ assay (≥0.1) status as the dependent variable (SCITT/ IFN-γ: -/+ = 1; -/- = 0).
2. IFN-γ assay-negative and SCITT-positive (Figure 2).
c. Model c): Subset of IFN-γ assay (<0.1)-negative animals, using SCITT (>2 mm) status as the dependent variable (IFN-γ/SCITT: -/- = 0; -/+ = 1).
d. Model d): Subset of SCITT (>2 mm)-positive animals, using IFN-γ assay (<0.1) status as the dependent variable (SCITT/ IFN-γ: +/- = 1; +/+ = 0).

A number of two subsets of each type of disagreement between the two diagnostic tests were used to produce four disagreement models. The outcome variable was the binary result for the alternative test with the result of interest being the contrary binary result, e.g., subset positive and outcome variable negative. Host-related factors (e.g., age, sex, breed, and evidence of co-infections) were included. Model selection was performed through model averaging; this approach allowed us to generate, rank, and weight several models supporting hypothesised associations to the outcome of interest (disagreement) (51). A set of explanatory variables were included in the global model for each disagreement, and this was the starting point to generate a model set to compare and average. Submodels were generated through the *dredge* function implemented in the MuMIn package (52). Each submodel was assessed by its AIC (53), and submodels with a Δ AIC ≤ 2 were averaged (54). For all models, herd was included as a random effect. Receiver operating characteristic (ROC) curves were used to assess the capacity of the models to discriminate between agreements and disagreements.

## Results

### Cattle Sample

In total, 100 pastoral herds were recruited, 50 in the NWR and 50 in the VIN, with 14–15 cattle sampled per herd. Complete dataset for both tests was available for 750 cattle in NWR and 741 cattle in VIN (*n* = 748 for IFN-γ assay; *n* = 741 for SCITT). A detailed summary of animal (55) and herd-level data (29) has been previously published. This study utilises the animal-level dataset, accounting for herd and study site in the analysis where appropriate.

### Bovine Tuberculosis Diagnostic Test Agreement

**Figure 3:**
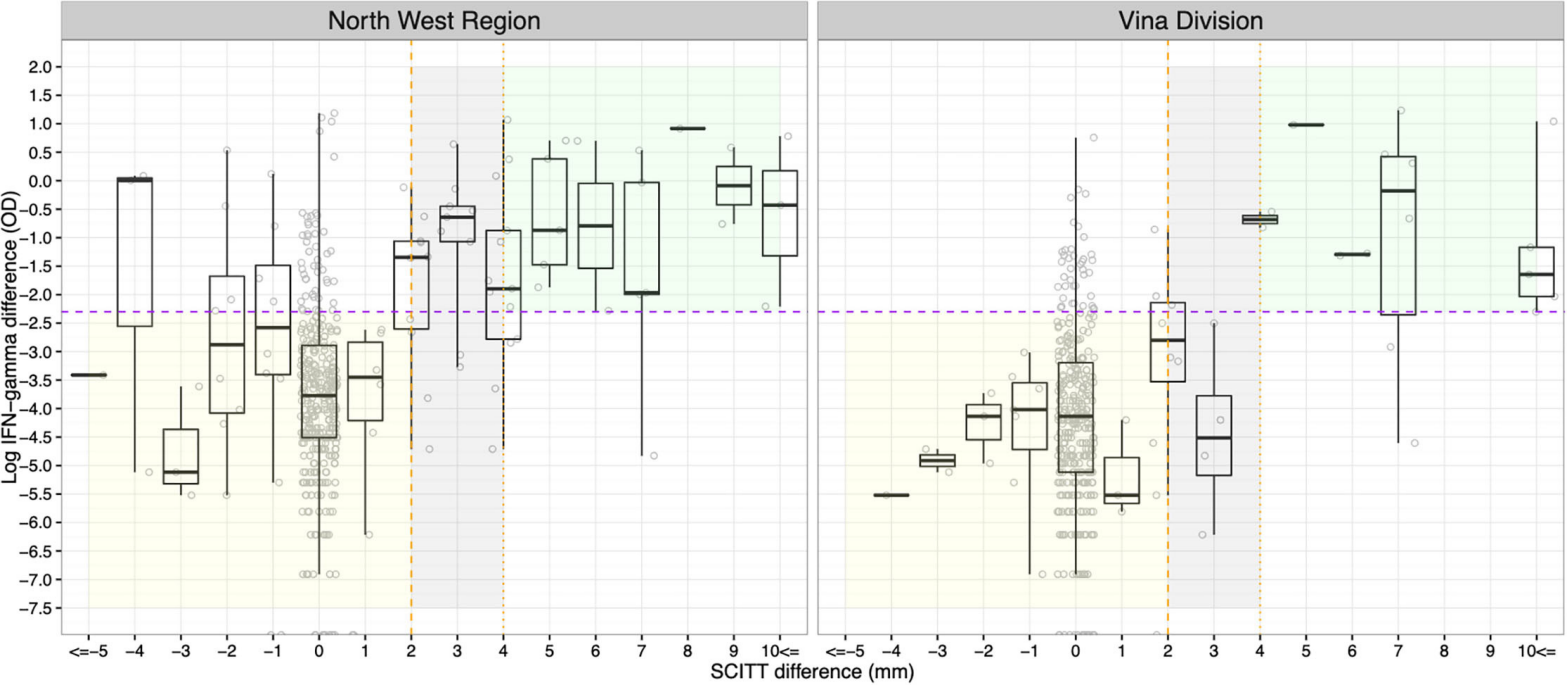
For the IFN-γ assay, results are displayed on a log scale for clarity and ≥0.1 positive cut-off values are shown (horizontal purple dashed line; ln (0.1) = −2.3). For the SCITT >2 mm (vertical orange dashed line) and >4 mm (vertical orange dotted line), positive cut-off values are shown. The green area denotes the test positive cattle for IFN-γ assay (≥0.1) and SCITT (>4 mm). The grey area denotes proportion of additional test positive cattle for IFN-γ assay (≥0.1) and SCITT (≥2 mm). The yellow area denotes test negative cattle for IFN-γ assay (<0.1) and SCITT (≤ 2 mm).

The raw continuous results for the SCITT (difference in skin thickness in mm) and IFN-γ assay (OD difference) stratified by site are presented in Figure 3. The frequency of avian PPD greater than bovine PPD reactions for SCITT (at >2 mm: 1.25%, 95% CI: 0.79–1.97%; at >4 mm: 0.01%, 95% CI: 0.00–0.39%) and IFN-γ (at ≥0.1: 7.11%, 95% CI: 5.90–8.55%) was low. The agreement between the binary two test results, with the SCITT at >2 mm and >4 mm positive cut-off values, is presented in Table 1. The percentage agreements were relatively high but do not adjust for agreement by chance and because of the low apparent prevalence can be misleading, and thus, the Cohen's κ statistic gives a better measure adjusting for chance. The agreement at >2 mm cut-off was slightly higher for the NWR compared to at >4 mm cut-off, but the different cut-offs were identical for the VIN. The scores were relatively low suggesting fair-moderate agreement for the NWR but poor-moderate agreement for the VIN. As the confidence interval for the Cohen's κ statistic overlapped for estimates between the study sites, and previous studies highlighting the use of >2 mm positive cut-off (30), the SCITT >2 mm positive cut-off value was chosen for the remainder of the analysis.

**Table 1:** Comparisons of agreement and Cohen's κ statistic between IFN-γ assay (≥0.1) and SCITT (>2 mm and >4 mm) for pastoral cattle sampled in the North West Region and Vina Division.

**North West Region (*****n*** **=** **750)**					
IFN-γ assay (≥0.1)	SCITT (>2 mm)			Percentage agreement	Cohen's κ statistic (95% CI)
		+	-	90.8%	0.42 (0.31–0.53)
	+	30	55		
	-	14	651		
IFN-γ assay (≥0.1)	SCITT (>4 mm)			Percentage agreement	Cohen's κ statistic (95% CI)
		+	-	90.5%	0.28 (0.17–0.39)
	+	16	69		
	-	2	663		
**Vina Division (*****n*** **=** **741)**					
IFN-γ assay (≥0.1)	SCITT (>2 mm)			Percentage agreement	Cohen's κ statistic (95% CI)
		+	-	93.7%	0.33 (0.18–0.47)
	+	13	36		
	-	11	681		
IFN-γ assay (≥0.1)	SCITT (>4 mm)			Percentage agreement	Cohen's κ statistic (95% CI)
		+	-	94.5%	0.33 (0.18–0.47)
	+	11	38		
	-	3	689		

### Bovine Tuberculosis Apparent Prevalence Estimates

**Figure 4:**
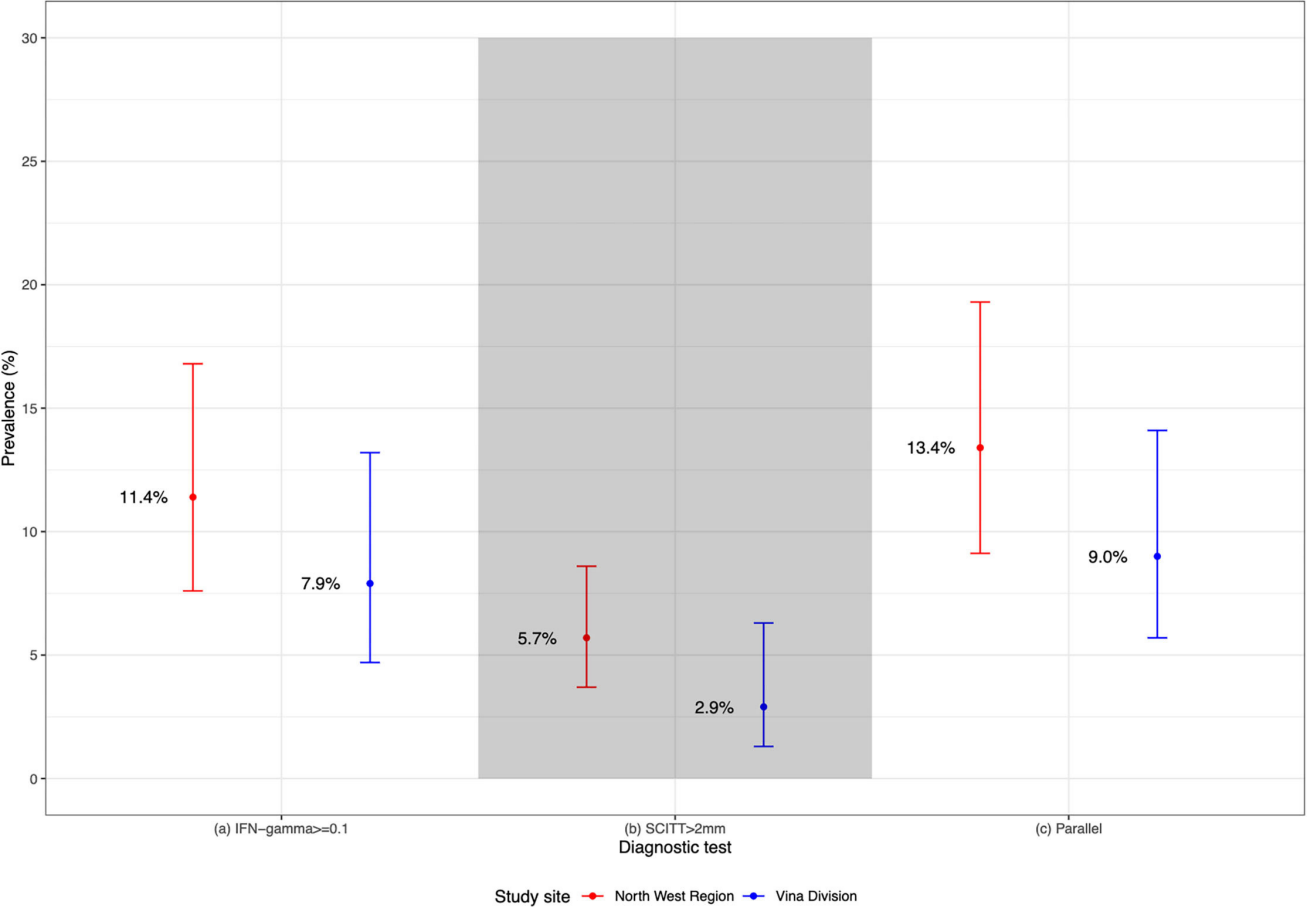
Summary of apparent bTB prevalence, using IFN-γ assay (≥ 0.1), SCITT (>2 mm) and parallel combination testing. IFN-γ assay (≥ 0.1) (NWR *n* = 750; VIN *n* = 748); SCITT (>2 mm) and Parallel (NWR *n* = 750; VIN *n* = 741).

**Figure 5:**
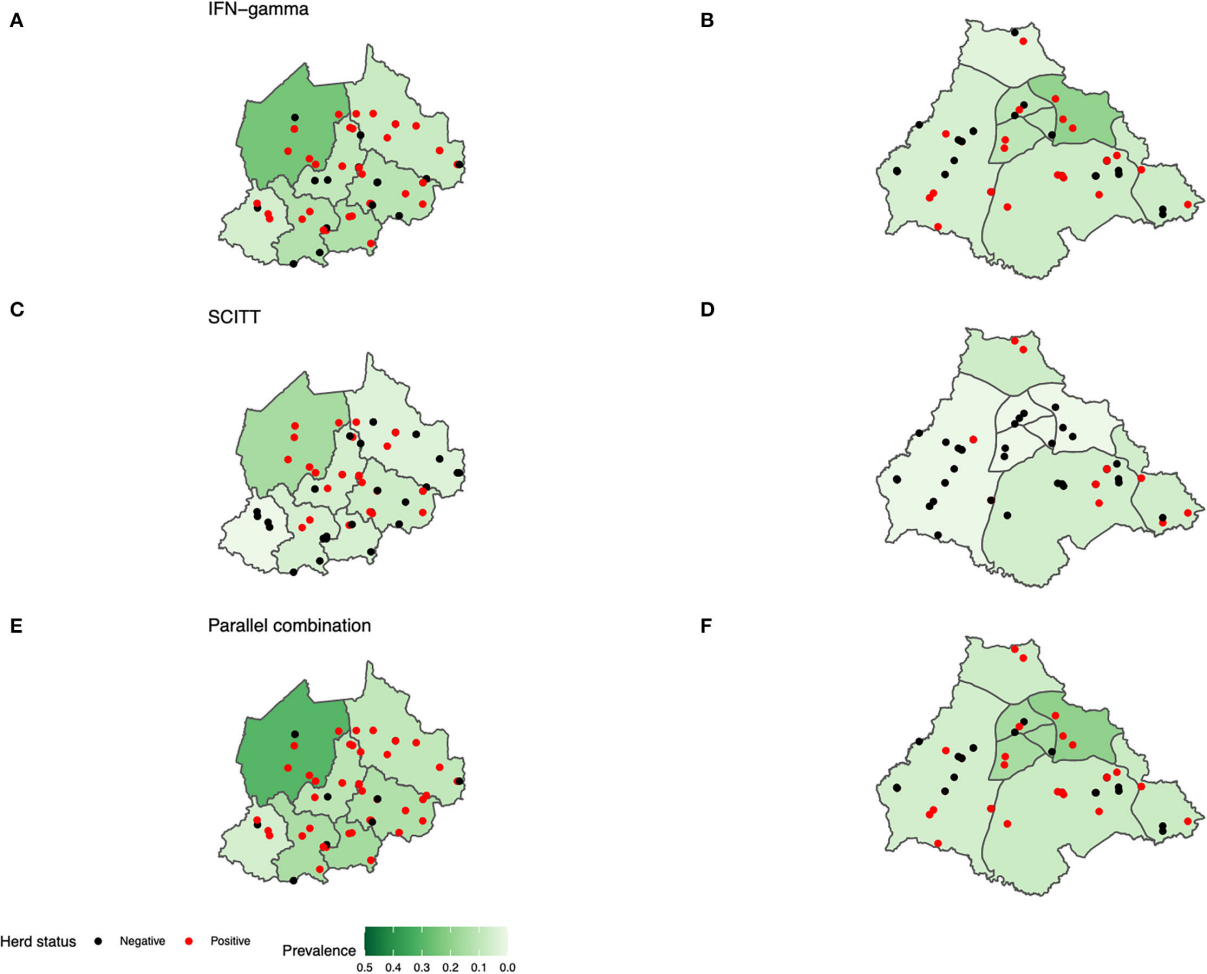
Map study site by sublocation prevalence for pastoral cattle. **(A,B)** IFN-γ assay (≥0.1), **(C,D)** SCITT (>2 mm) and **(E,F)** Parallel combination [subfigure **(A,C,E)** NWR *n* = 750. **(B)** VIN *n* = 748. **(D,F)** VIN *n* = 741]. Positive herds have at least one infected animal per herd.

The apparent prevalence estimates between the NWR and VIN are compared in Figures 4, 5; based on the IFN-γ assay (≥0.1), SCITT (>2 mm) and a parallel combination (PC) of the two tests (i.e., positive on either or both tests at the specified cut-offs). The highest estimates were generated when a parallel approach is used, followed by the IFN-γ and SCITT. No significant difference in overall animal-level apparent prevalence was noted between study sites when using any single or PC tests, although the prevalence was consistently lower in the VIN than the NWR (Figure 4). The different tests give quite a different impression of the magnitude in apparent prevalence across administrative areas and herds that are defined as positive (at least 1 positive animal per herd) (Figure 5).

### Risk Factors Associated With Bovine Tuberculosis Diagnostic Test Disagreement

#### IFN-γ Assay-Positive and SCITT-Negative

Results from sampled cattle were subsetted to investigate diagnostic disagreement for being IFN-γ assay-positive and SCITT-negative using two methods to produce two final models (Figures 2A,B described the disagreement investigated using the models outlined Table 2). The final models are presented in Table 2. Model a looked at animals that were IFN-γ-positive and then looked at factors associated with testing SCITT-negative (*n* = 133). Only age was strongly associated with disagreement. In other words, older animals were more likely to test negative on the SCITT test given they were IFN-γ-positive. Model b looked at the reverse and looked at SCITT-negative animals and factors associated with them being positive for the IFN-γ (*n* = 1422). Here, female animals were less likely to be IFN-γ-positive compared to males, and also interestingly, animals having had exposure to BLV or paraTB were more likely to be in disagreement. The area under the ROC curve for the final averaged models was 0.94 (95% CI: 0.90–0.97) and 0.87 (95% CI: 0.85–0.91) for models a and b, respectively. This was an indicative of good model performance in relation to classifying the disagreements.

**Table 2:** Final models to investigate risk factors for pastoral cattle testing IFN-γ assay-positive and SCITT-negative.

**Model a (*n* = 133)**	**Global model: SCITTdiff2~AGE2** **+** **ANISEX** **+**	
**11 models**	**ABREED2** **+** **FgLivB** **+** **BVDAbPN**	
	**+** **paraAbPN** **+** **strata1** **+** **(1|HER_ID)**	
	***Binary outcome: SCITT-negative***.	
Variable	Level	OR (95% CI)
Age	Young	Reference
	Adult	7.57 (1.69–33.84)
	Old Adult	7.21 (1.65–31.54)
**Model b (*****n*** **=** **1422)**	**Global model: bovigam01~AGE2** **+** **ANISEX**	
**9 models**	**+** **ABREED2** **+** **FgLivB** **+** **LVAbPN** **+** **BVDAbPN**	
**+** **paraAbPN** **+** **strata1** **+** **(1|HER_ID)**		
***Binary outcome: IFN-*****γ*****-positive***.		
Variable	Level	OR (95% CI)
Sex	Male	Reference
	Female	0.50 (0.31–0.83)
Enzootic Bovine Leucosis	Negative	Reference
	Positive	2.30 (1.04–5.05)
Paratuberculosis	Negative	Reference
	Positive	6.54 (2.57–16.61)

*Two models investigate diagnostic disagreement: (model a) Dependent variable SCITT-negative (SCITTdiff2) in IFN-γ-positive subgroup (n = 133). (model b) Dependent variable IFN-γ assay-positive (bovigam01) in SCITT-negative subgroup (1422). Explanatory variables included are AGE2 (age: young, adult or old adult), ANISEX (sex: female or male), ABREED2 (breed: improved or Fulani), FgLivB (F. gigantica serology result: negative or positive), LVAbPN (bovine leucosis virus serology: negative or positive), BVDAbPN (bovine viral diarrhoea virus serology result: negative or positive), paraAbPN (Paratuberculosis serology: negative or positive), strata1 (study site) and random effect HER_ID (herd sampled from)*.

#### IFN-γ Assay-Negative and SCITT-Positive

Cattle were also subsetted to investigate diagnostic test disagreement for being SCITT-positive and IFN-γ assay-negative using two methods (Figures 2C,D described the disagreement investigated using the models outlined Table 3). The final models are presented in Table 3; model c looked at animals that were IFN-γ-negative and then looked at factors associated with testing SCITT-positive (*n* = 1,364). Only age was strongly associated with disagreement, where animal that has tested negative on the IFN-γ it is more likely to be SCITT-positive if it is an older animal (adult or older adult) compared to the young animal. For model d, using the subset of animals testing SCITT-positive, there were no factors strongly associated with animals testing IFN-γ-negative (disagreement), but the sample size for this subset was very small (*n* = 68). The area under the ROC curve for the final averaged models was 0.90 (95% CI: 0.83–0.96) and 0.99 (95% CI: 0.97–1.00) and for models c and d, respectively. These results indicate that the models correctly classified the disagreements.

**Table 3:** Final models to investigate risk factors for pastoral cattle testing IFN-γ assay-negative and SCITT-positive.

**Model c (*n* = 1364)**	**Global model: SCITTdiff2~ANISEX** **+** **ABREED2**	
**7 models**	**+** **FgLivB** **+** **LVAbPN** **+** **BVDAbPN** **+** **AGE2** **+** **strata1**	
	**+** **(1|HER_ID)** ***Binary outcome: SCITT-positive***.	
Variable	Level	OR (95% CI)
Age	Young	Reference
	Adult	15.74 (2.10–120.20)
	Old Adult	9.18 (1.12–75.41)
**Model d (*****n*** **=** **68)**	**Global model: bovigam01** **~ANISEX** **+** **ABREED2**	
**8 models**	**+** **FgLivB** **+** **BVDAbPN** **+** **LVAbPN** **+** **strata1**	
**+(1|HER_ID)** ***Binary outcome: IFN-*****γ*****-negative***		
Variable	Level	OR (95% CI)
Nothing significant.		

*Two models investigate diagnostic disagreement: (c) Dependent variable SCITT-positive (SCITTdiff2) in IFN-γ-negative subgroup (n = 1,364). (d) Dependent variable IFN-γ assay-negative (bovigam01) in SCITT-positive subgroup (68). Explanatory variables included are AGE2 (age: young, adult or old adult), ANISEX (sex: female or male), ABREED2 (breed: improved or Fulani), FgLivB (F. gigantica serology result: negative or positive), LVAbPN (bovine leucosis virus serology: negative or positive), BVDAbPN (bovine viral diarrhoea virus serology result: negative or positive), paraAbPN (paratuberculosis serology: negative or positive), strata1 (study site) and random effect HER_ID (herd sampled from)*.

## Discussion

In this study, we highlight that the use of the IFN-γ assay, SCITT, and parallel combination tests can give a quite different impression of the overall magnitude of bTB prevalence in a naturally infected cattle population. Although individual or combined test prevalence estimates overlapped in the two localities, the lowest and highest estimates in each locality differed by multiples of ~2–3. Internationally, the individual and combined use of IFN-γ assay and SCITT are used to estimate prevalence to prioritise local resources in bTB surveillance and control programmes (2, 3). Inaccurate estimates of bTB prevalence will likely have a detrimental public health impact where resources for control are limited. This is of particular concern in SSA contexts where *M. bovis* is undoubtedly a public health issue, due to a close interaction between cattle and humans, and disease surveillance is infrequent. Understanding the level of agreement and host factors associated with disagreement will improve interpretation of future bTB prevalence estimates in SSA cattle populations.

Although the antemortem tests measure the CMI response to *M. bovis* infection (56), and a degree of agreement is likely, agreement between the two tests was only “moderate” [κ = 0.42 (moderate agreement: 0.41–0.60)] at best when using positive cut-off values of ≥0.1 and >2 mm positive cut-offs for the IFN-γ assay and SCITT, respectively. Even lower agreement was noted between the IFN-γ assay and the SCITT was reported at >4 mm, defined as poor [κ = 0.13 (poor agreement: 0.01–0.2)]. Poor agreement has also been reported between the two assays in Ethiopia where the >4 mm cut-off was used for the SCITT and ≥0.1 for the IFN-γ assay (57). Other studies in SSA, including Cameroon, reported that bTB-positive *B. indicus* cattle respond differently to *M. bovis* PPD, when compared to *B. taurus* cattle, with improved agreement when using the >2 mm cut-off for the SCITT (58–60). Suggesting that use of a lower >2 mm cut-off for the SCITT could be more appropriate in cattle populations in SSA. The IFN-γ assay and the SCITT also do not identify the same bTB positive population of cattle, possibly because the two tests are measuring different aspects of the CMI response (15, 61). Sampled cattle were from a naturally infected population, where *M. bovis* infected cattle are likely infected at different time points, likely to be at different stages in pathogenesis and consequently have marked variation in their host immune responses at the time of sampling. The IFN-γ assay is considered to be more sensitive than the SCITT and can detect *M. bovis* infection weeks–months earlier than the SCITT test (10, 62), which could explain why in our study, the IFN-γ assay consistently detecting more positives than the SCITT (at either study site). This suggests that potentially, the IFN-γ assay may be more suitable for highlighting the potential magnitude of prevalence in resource-limited settings, by detecting animals earlier post-infection. Further work is required to understand the diagnostic performance of the IFN-γ assay compared to the SCITT to improve the accuracy of prevalence estimates in naturally infected cattle populations.

Using subsets of the complete dataset, of test positive and negative animals, we were able to explore host-related factors for disagreement. Being IFN-γ assay-positive and SCITT-negative was a far more common form of disagreement and, as reported in other studies (16, 63–66), potentially associated with the low sensitivity of the SCITT, although reasons for reported lower sensitivity of the SCITT are poorly eluded. The impact of co-infections is likely to depend on pathogen or subtype exposed to, burden and timing of exposure (7, 18, 67). Bovine leucosis virus and paratuberculosis positivity were reported to increase the risk of this form of disagreement. As in our study, animals sampled from naturally infected populations may be co-infected with immunosuppressing or modulating infections and have been reported to influence CMI immune responses (7). Exposure to bovine leucosis virus can result in a persistent immunosuppression which may lead to depression in CMI responses, although its less clear why one CMI diagnostic may be affected more than another. Non-tuberculous mycobacteria have been shown to infect Cameroonian cattle (34) yet as both CMI diagnostic tests included a control avian PPD in their protocols, and the impact of non-tuberculous mycobacteria is likely to vary (68–70). Other researchers have identified that the performance of both the SCITT and the IFN-γ assay can be affected by paratuberculosis (*Mycobacterium avium* subspecies *paratuberculosis*) positivity in experimental infections (28, 71–73) specifically with increased reactivity to avian PPD. Although it is currently unclear why the IFN-γ-positive/ SCITT-negative disagreement was predominant in our study, this might be related to younger animals being less impacted by MAP immunosuppressive responses than older animals (>1 year of age) (74). Although frequency of avian PPD and paratuberculosis positives was low in this study, the addition of mitogen to IFN-γ assay may be helpful to improve diagnostic sensitivity (13, 75) when testing Cameroon or similar SSA cattle populations. Interestingly, *F. gigantica* exposure was not associated with increased risk of disagreement between the CMI diagnostics. This is contra to an abattoir study, also conducted by authors, which demonstrated that *F. gigantica* was associated with decreased IFN-γ positivity and larger bTB lesions (23). Although it is unclear why a similar relationship was not also noted in this field study, multiple parasite and host factors are likely to affect the interaction between *M. bovis* and *F. gigantica* at any given time point. For example, experimental studies have demonstrated that a related *Fasciola* species, *Fasciola hepatica*, has been associated with decreases in diagnostic sensitivity for IFN-γ and SCITT when compared to detection of bTB lesions postmortem (76), but the extent of the effect on bTB diagnosis is determined by the order of infection of the two organisms (76). In our study, fluctuations in exposure or burden are not captured by the *F. gigantica* antibody ELISA (35) as the test detects exposure to infection at some point in an animal's lifetime. Future work should focus on investigating the variation in the bovine immune response to *M. bovis* in naturally infected cattle beyond singular time points and the dynamic impact of co-infections on bTB diagnostic test performance.

Advanced age was reported to be associated with both types of disagreement (IFN-γ-positive/ SCITT-negative and IFN-γ-negative/ SCITT-positive). In later stages of infection, IFN-γ responses can fluctuate throughout the course of *M. bovis* infection and may lead to IFN-γ assay false negatives (56, 65) when SCITT is positive. Furthermore, IFN-γ responses become anergic in chronic *M. bovis* infections (56), and advanced age of cattle could be a proxy for chronicity when investigating disagreement. IFN-γ assay-negative and SCITT- positive disagreement were less frequently reported which is potentially due to the higher specificity of the SCITT (16, 63–65). Cattle with chronic *M. bovis* infections are thought to be more likely to become SCITT-positive if they initially start out as IFN-γ-positive (12, 77), which may partly explain why this form of disagreement occurred less frequently.

In the absence of a gold-standard diagnostic for bTB, we were unable to fully explore the diagnostic performance of animals sampled using antemortem diagnostic tests. However, we were able to explore the associations with host factors that may account for diagnostic disagreement, using multivariable mixed-effects logistic regression modelling and a model averaging selection method. Compared to traditional methods of model selection, model averaging techniques allowed us to better identify the factors associated with the disagreements and the risk factors through a better estimation of the coefficients. Similar to Bayesian approaches, several models can be ranked and weighted to provide a quantitative measure of relative support for each competing hypothesis (51). By comparison, more traditional approaches such as stepwise methods, although also resulting in a final model, completely ignore model uncertainty (78). It is clear that co-infections can have complex impacts on bTB test diagnostics performance, and this may have important implications for bTB prevalence estimates along with future surveillance and control programmes in SSA. Although we did not test for every co-infection possible, our study has highlighted the need to consider animal level factors when interpretating bTB diagnostic test results to develop accurate prevalence estimates in cattle populations of interest.

## Conclusion

Inaccuracies in local prevalence estimates hinder the progress of bTB surveillance and control programmes. In this study, we demonstrated that individual or combined use of the IFN-γ assay and SCITT can lead to a large variation in bTB prevalence estimates. Animal-level factors may impact on the agreement between CMI diagnostics and could limit our understanding of bTB epidemiology in endemic settings, where animals of various disease states exist. Quantifying the impact of factors, such as co-infections, should be prioritised to improve the accuracy of diagnosis and understanding of bTB epidemiology across cattle populations in SSA as well as other LMICs.

## Data Availability Statement

The raw data supporting the conclusions of this article will be made available by the authors, without undue reservation.

## Ethics Statement

The study was reviewed and approved by the University of Edinburgh Ethics Committee, UK (ERC No: OS02-13) and by the Institute of Research and Development (IRAD), Cameroon. All participants gave informed verbal consent to the translator before participating and could opt out at any stage. Written informed consent for participation was not required for this study in accordance with the national legislation and the institutional requirements.

## Author Contributions

BB, LN, and VT conceived the original project. BB, RK, KM, LN, VT, MS, and NE designed the field study, the databases, and the survey instrument. RK, NE, VN, VT, KM, and BB developed the field SOPs and collected the data. RK, EF, and LG conducted laboratory work. RK and BB cleaned the initial dataset. RK, LG, BB, and IH contributed to the analysis. RK was responsible for writing the initial drafts. All authors contributed comments for the final draft. All authors contributed to the article and approved the submitted version.

## Funding

This work was funded by the Wellcome Trust (WT094945).

## Conflict of Interest

The authors declare that the research was conducted in the absence of any commercial or financial relationships that could be construed as a potential conflict of interest.

## Publisher's Note

All claims expressed in this article are solely those of the authors and do not necessarily represent those of their affiliated organizations, or those of the publisher, the editors and the reviewers. Any product that may be evaluated in this article, or claim that may be made by its manufacturer, is not guaranteed or endorsed by the publisher.
